# Hepatocellular Carcinoma in Nonalcoholic Fatty Liver Disease

**DOI:** 10.7759/cureus.754

**Published:** 2016-08-29

**Authors:** Faisal Inayat, Zia Ur Rahman, Abu Hurairah

**Affiliations:** 1 Department of Medicine, New York-Presbyterian Hospital, Weill Cornell Medical College, New York City, NY, USA; 2 Department of Internal Medicine, East Tennesse State University; 3 Division of Gastroenterology, Department of Medicine, SUNY Downstate Medical Center, Brooklyn, NY, USA

**Keywords:** hepatocellular carcinoma, nonalcoholic fatty liver disease, non-cirrhotic, prevalence, surveillance

## Abstract

Our objective was to study nonalcoholic fatty liver disease (NAFLD) as a relevant risk factor associated with hepatocellular carcinoma (HCC) in patients with and without cirrhosis. HCC is a common cancer worldwide that predominantly involves patients with hepatic cirrhosis. HCC has recently been linked to NAFLD, the hepatic manifestation of obesity and related metabolic disorders. This association is alarming due to the high prevalence of NAFLD globally, which may contribute to the rising incidence of HCC.

A 31-year-old female with a history of dyslipidemia, hypertension, and diabetes mellitus presented with abdominal pain that persisted for six months. The pain was associated with gastrointestinal symptoms and weight loss. She was drug-free and a nonalcoholic and a nonsmoker. The physical examination was unremarkable. The abdominal exam showed a soft and non-tender abdomen, with no organomegaly or ascites. The laboratory evaluation was unremarkable. The imaging studies showed a hypodense lesion in the right hepatic lobe with strong arterial enhancement. Subsequently, the patient underwent a liver biopsy. The histopathology results were consistent with HCC.

The patient underwent an uneventful segment VI liver resection and tumor-free margins were achieved. In our patient, NAFLD was designated as an independent etiology for HCC, without cirrhosis. Our patient recovered well and has been disease free for over a year. HCC may complicate non-cirrhotic NAFLD with mild or absent fibrosis, greatly expanding the population potentially at higher risk of HCC. These results provide new targets for surveillance, prevention, early recognition, and effective treatment of HCC associated with NAFLD.

## Introduction

Hepatocellular carcinoma (HCC) is the second leading cause of cancer-related deaths worldwide [1]. Recently, HCC has been designated as a complication of nonalcoholic fatty liver disease (NAFLD) [1]. This association of NAFLD and HCC has been supported by multiple small observational studies, which suggest age and advanced fibrosis as significant risks [1-2].

We present a young, nonalcoholic, female patient with a history of dyslipidemia, hypertension, and diabetes mellitus diagnosed with HCC on a standard set of investigations. The patient had no evidence of hepatic fibrosis or cirrhosis. Early detection led to the effective treatment of HCC in our patient, and curative resection was performed in a timely manner. Informed consent was obtained from the patient for this study.

The prevalence of NAFLD is increasing with the growing epidemics of diabetes and obesity. It is imperative that the pathogenesis of NAFLD-induced HCC should be studied in larger randomized controlled trials in the non-cirrhotic patient population.

Hence, surveillance and prevention protocol formulation will have immense diagnostic importance for this probable duo, especially for people with obesity and metabolic syndromes. It will help to reduce the number of cases of HCC secondary to NAFLD and will have an impact on the tremendously increasing morbidity and mortality following HCC.

## Case presentation

A 31-year-old female presented to the SUNY Downstate Medical Center Emergency Department with abdominal pain that persisted for six months. It was a waxing and waning right upper quadrant pain (RUQ) that worsened over three to four weeks prior to the presentation. The pain was associated with nausea, vomiting, diarrhea, subjective fevers, and a weight loss of _~_20 lbs. Her past medical history was significant for dyslipidemia, hypertension, and insulin-dependent diabetes mellitus, and she had been on regular medications. The patient had recently returned from a trip to Mexico. She was nonalcoholic and denied smoking or illicit drug use.

The physical examination was unremarkable. The abdominal exam showed a soft, non-tender abdomen with no organomegaly or free fluid. The laboratory studies revealed the following: a white cell count of 7800/uL (normal, 3,500–10,500/uL), hematocrit 38% (normal, 38.8–50.0%), hemoglobin 12 g/dL (normal, 12.0–15.5 g/dL), platelets 413000/uL (normal, 150,000–450,000/uL), albumin 4.2 g/dL (normal, 3.5–5.5 g/dL), total bilirubin 0.4 mg/dL (normal, 0.3–1.2 mg/dL), aspartate aminotransferase (AST) 16 IU/L (normal, 5–40 IU/L), alanine transaminase 22 IU/L (normal, 10–40 IU/L), serum alkaline phosphatase 81 IU/L (normal, 32–92 IU/L) and alpha-fetoprotein 2.7 ng/mL (normal, 0–8.5 ng/mL) with an international normalized ratio (INR) of 1.0 (normal, 2.0–3.0). Alpha-1 antitrypsin was 122 mg/dL (normal, 100–300 mg/dL). 

**Figure 1:**
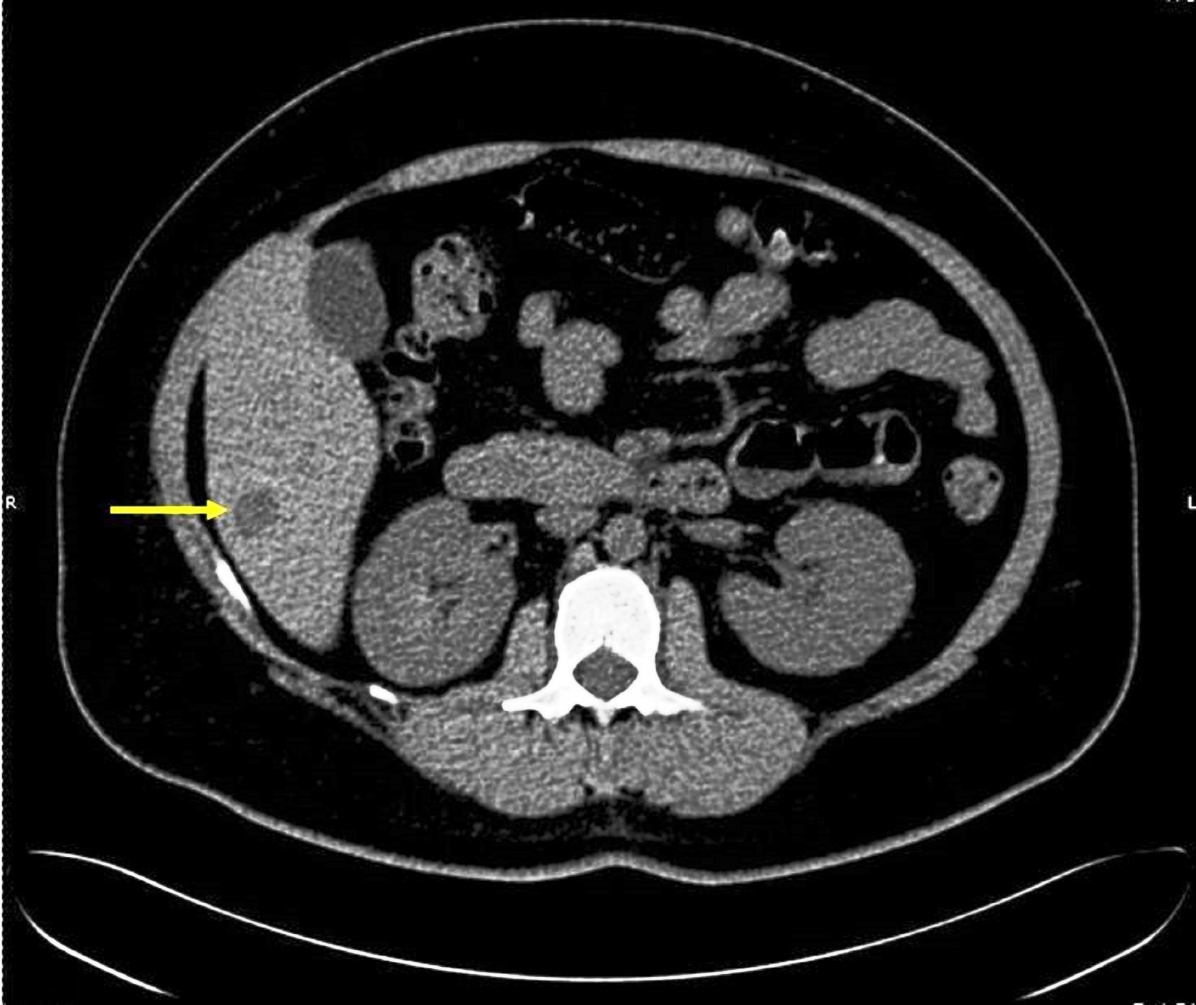
CT of the abdomen A CT scan of the abdomen without contrast showing a 1.6-cm hypodense lesion at the inferior aspect of the right hepatic lobe.

A computed tomography (CT) without contrast showed a relatively well-defined nodular mass of around 1.6 cm. It was a hypodense lesion at the inferior aspect of the right hepatic lobe (Figure 1).

**Figure 2:**
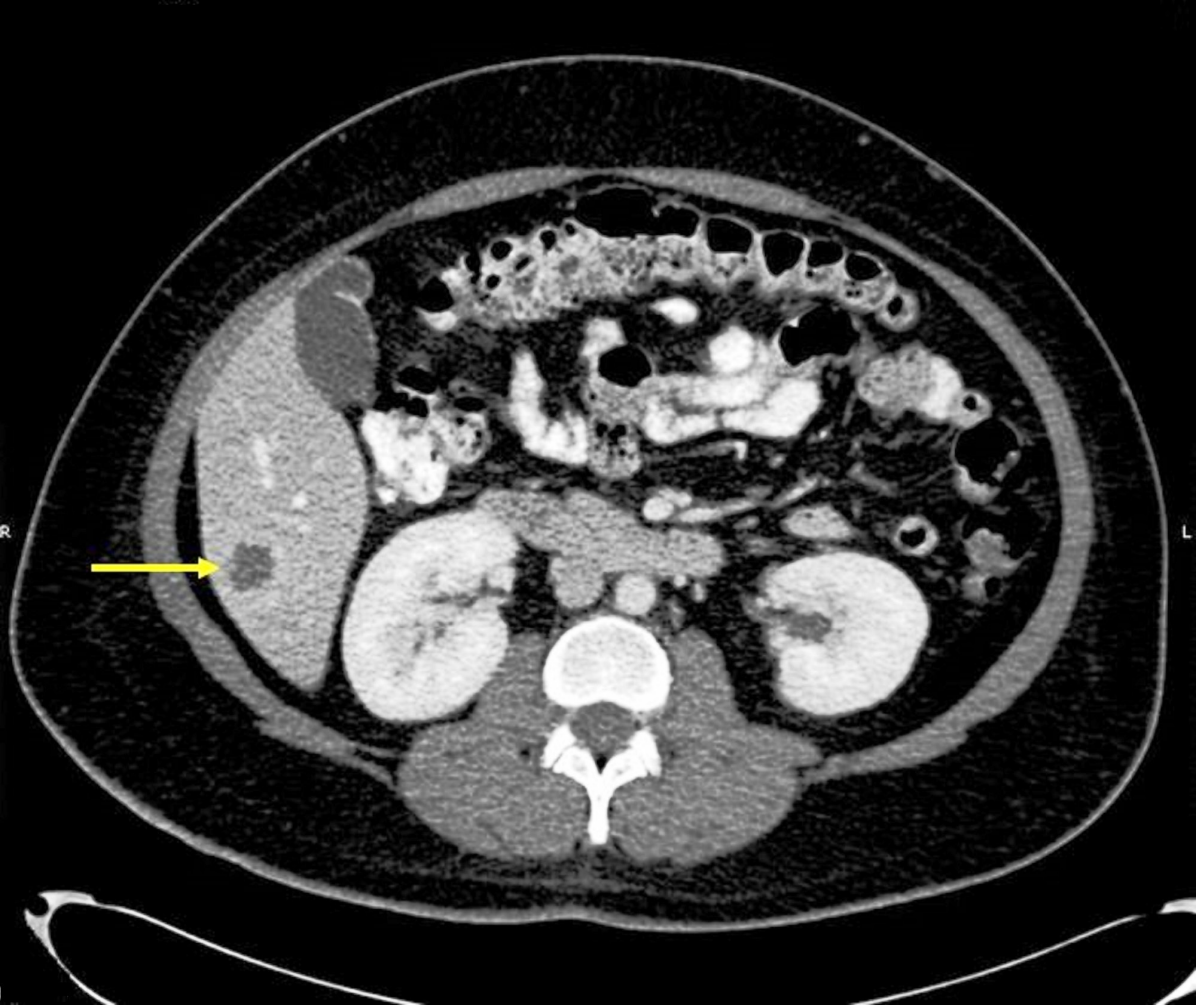
CT of the abdomen with contrast in arterial phase A hepatic segment VI 2.9 x 2.6 x 2.8 cm lesion demonstrating strong peripheral arterial enhancement with central isodense nature, which raised a concern for cystic change. However, all liver tumors get 100% of their blood supply from the hepatic artery, so when they enhance, it will be in the arterial phase.

A CT with contrast of the abdomen revealed a segment VI 2.9 x 2.6 x 2.8 cm lesion in the arterial phase. The lesion showed strong peripheral arterial enhancement. The central isodense nature raised concern for cystic change rather than necrosis (Figure 2).

**Figure 3:**
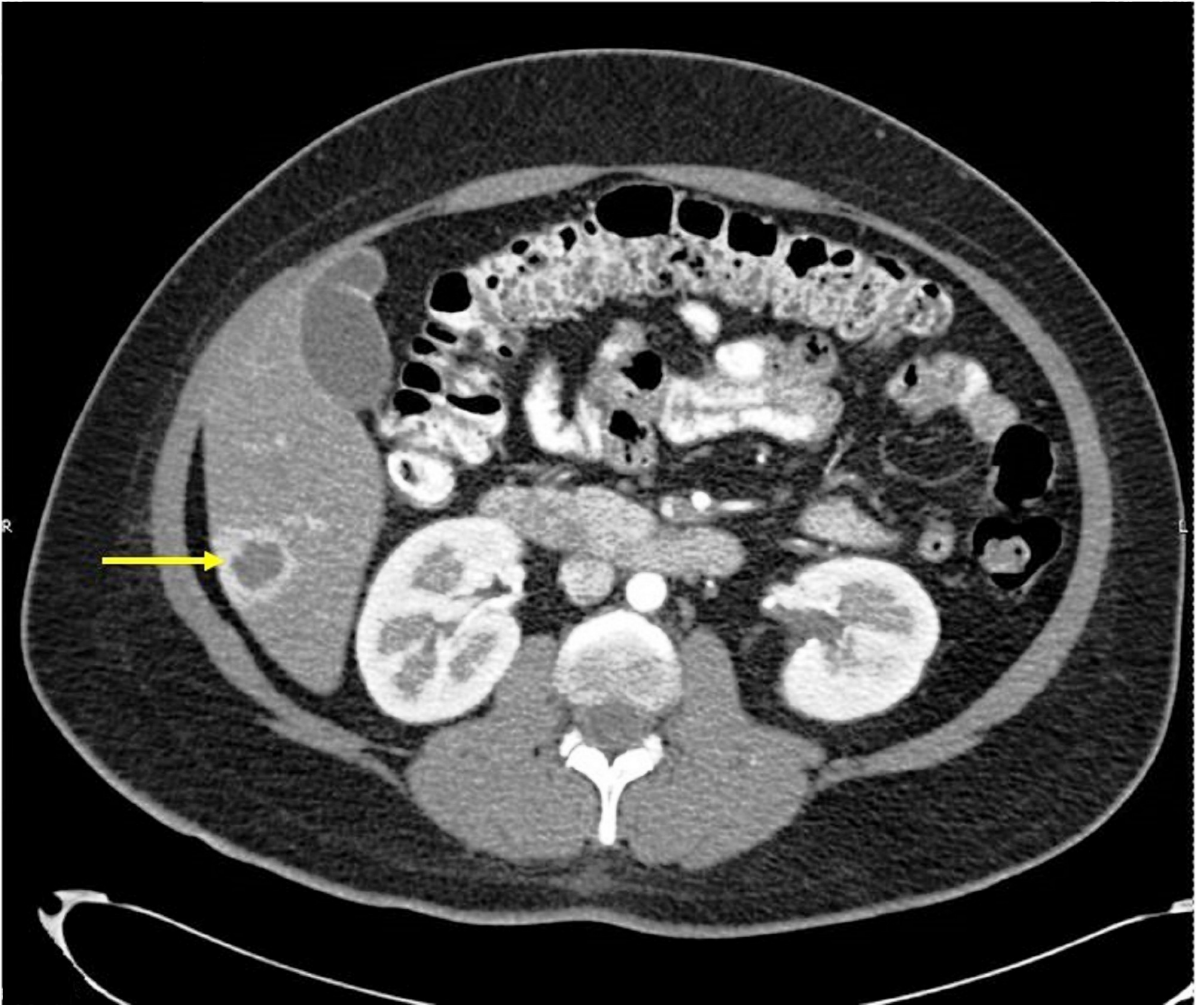
CT of the abdomen with contrast in venous phase The hepatic lesion became progressively isodense in venous phase.

The lesion became progressively isodense in venous and delayed contrast phases (Figure 3).

**Figure 4:**
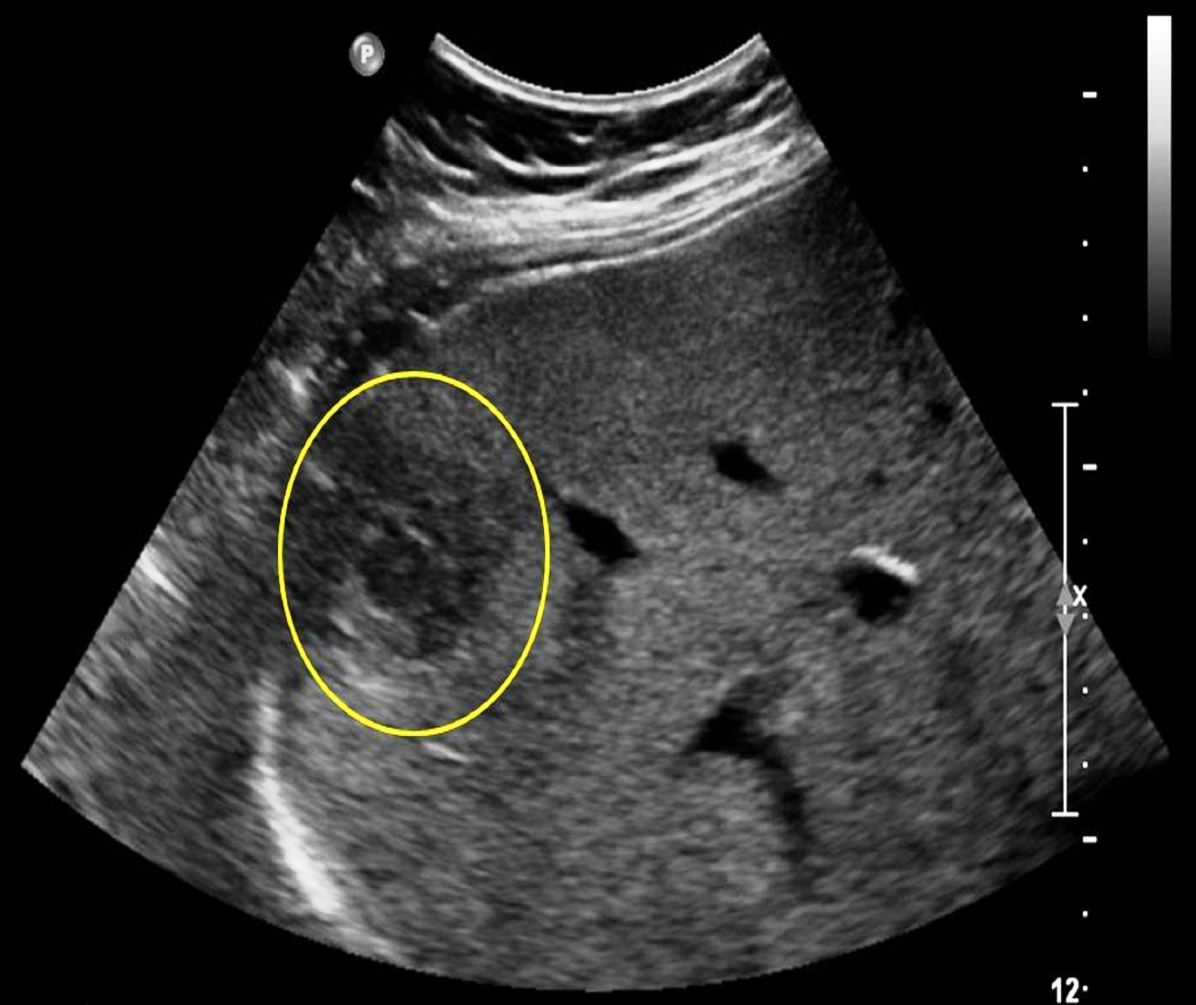
Ultrasonography RUQ A 15.4-cm lesion in the RMCL with a smooth contour and mildly heterogeneous echogenicity was identified. A 4.7 x 2.7 x 2.6 cm mostly hypoechoic, ovoid mass in the inferior aspect of the posterior right hepatic lobe contained a 1.6 x 1.1 x 1.3 cm focal hypoechoic structure with a mildly hyperechoic rim internally.

Ultrasonography demonstrated a mass with a smooth contour having mildly heterogeneous echogenicity with normal appearance in the right midclavicular line (RMCL) (Figure 4).

The color images showed increased blood flow along the periphery of the lesion. Therein, the patient underwent CT-guided fine needle aspiration and core liver biopsies with results suspicious for HCC. CD34 and reticulin special stain features confirmed the diagnosis of HCC.

**Figure 5:**
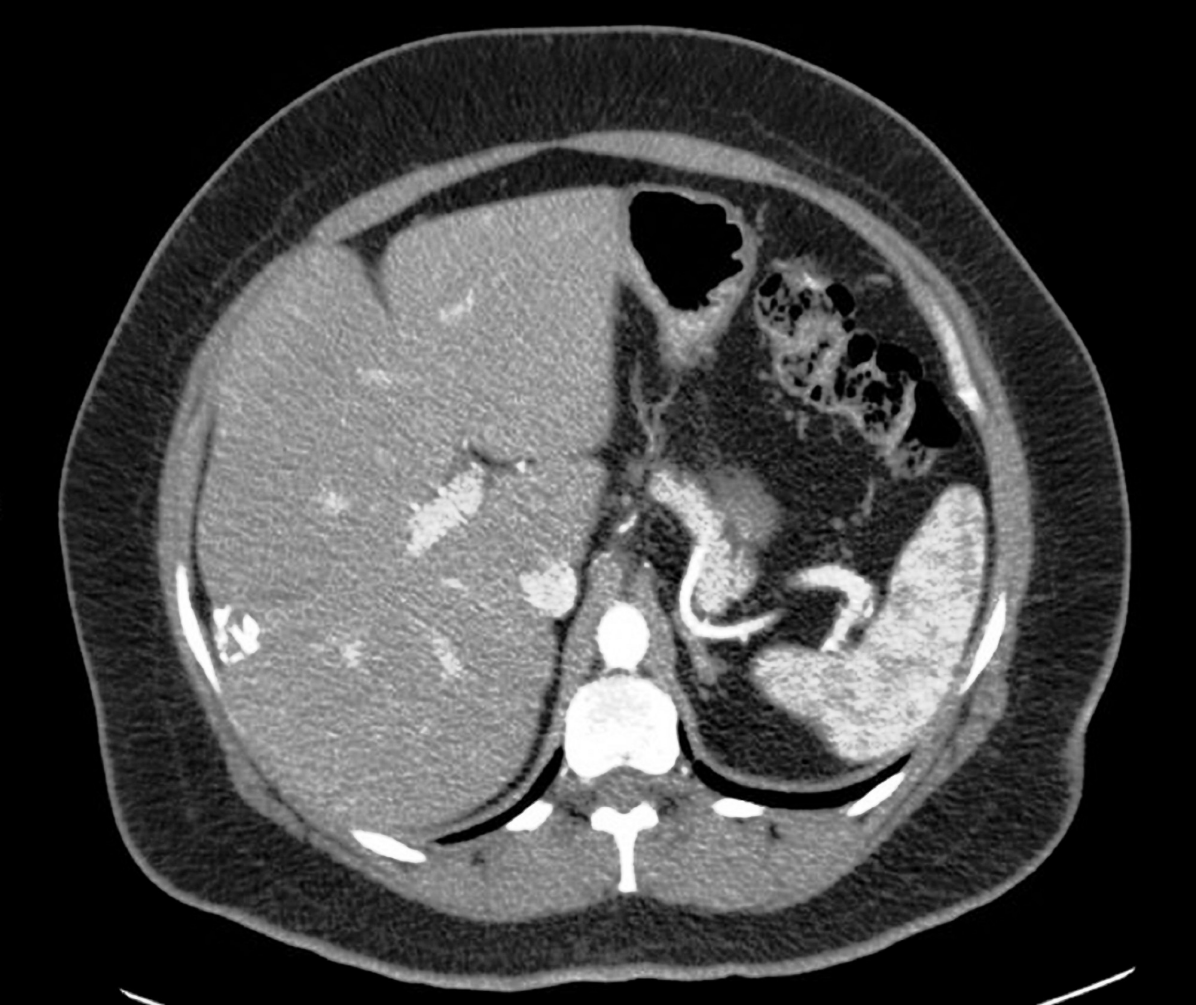
CT on follow-up after one year The previously treated lesion in segment VI did not reveal a significant change in size and had no enhancement.

Subsequently, the patient underwent an uneventful segment VI liver resection. The specimen was sent for histopathology, which demonstrated a well-differentiated HCC, 4.5 cm in greatest dimension with no lymphovascular invasion identified. Tumor-free resection margins were achieved (0.4 cm to the closest margin). The liver tissue revealed patchy steatosis (about 10%), mild clear cell degenerative changes, and mild non-specific chronic inflammation without significant fibrosis. NAFLD was designated as an independent etiology for HCC, without cirrhosis, in our patient. The patient had follow-up assessments of the treated lesion employing multiple calcific densities and clips after the hepatic wedge resection. The assessments did not reveal a significant change in size, and they showed no recurrence of the HCC for over a year (Figure 5).

## Discussion

HCC is a leading cause of cancer-related deaths worldwide. An increasing number of reports describe HCC in the setting of NAFLD. The increasing incidence of these conditions and the emerging evidence of HCC in non-cirrhotic NAFLD prioritize a better understanding of NAFLD-related HCC epidemiology and pathogenesis in order to target screening policies and develop preventive therapeutic strategies.

The pathogenesis of HCC in NAFLD is related to obesity and diabetes. It is believed that obesity plays an important role in HCC development by generating a chronic general low-grade inflammatory response due to an increased level of leptin and a relatively decreased level of adiponectin in obese patients [1]. Increased lipid storage in the liver leads to lipotoxicity that interferes with cellular signaling mechanisms and regulation of gene transcription promoting gene transcription alterations, which may result in HCC [1]. Furthermore, there is supporting evidence that compensatory hyperinsulinemia and insulin-like growth factor (IGF) in obesity may promote the development of HCC by activating various oncogenic pathways [1].

The alterations in gut microbiota may also affect the progression of NAFLD and the development of HCC in obese patients [1]. Furthermore, an increased iron absorption in nonalcoholic steatohepatitis (NASH) patients has been demonstrated, and iron deposition has also been related to HCC development in NAFLD-cirrhosis. The underlying mechanisms might be related to oxidative DNA damage [1], but further studies are required to better understand the role of iron accumulation in NAFLD and HCC. Genetic polymorphism might also play a role in the development of HCC in NAFLD [1].

The mechanisms of hepatocarcinogenesis in steatosis might be different from the classic mechanisms involved in cirrhosis, and this could explain the high number of reported HCC in non-cirrhotic NAFLD [2]. It has, in fact, been reported that a significant number of patients with NAFLD-related HCC have no extensive fibrosis at presentation. A recent multicenter study described that steatohepatitis was more prevalent in the HCC cohort in comparison to the cholangiocellular cohort that showed an incidence similar to the general population, suggesting that steatohepatitis could play an important role in the development of non-cirrhotic HCC. It raised the hypothesis that HCC in NAFLD may arise in the absence of histologically evident inflammation [3].

The malignant transformation of hepatocellular adenoma (HCA) in non-cirrhotic patients with NAFLD is another possibility to consider in such patients. A correlation between HCA’s malignant transformation and metabolic syndrome has been described in the literature [4]. These data suggest that NAFLD, as the hepatic manifestation of metabolic syndrome, may lead to the development of HCC in the setting of HCA without cirrhosis. However, additional epidemiological and pathophysiological data are needed to prove this correlation.

The present report and other studies in the series have numerous clinical implications. NAFLD is increasing in prevalence in the United States and other affluent countries due to an overall increase in obesity and metabolic syndrome rates in these nations. One recent prospective cohort study found the prevalence of NAFLD in middle-aged US patients to be 46% based on ultrasonography and liver biopsy results [5]. As NAFLD is a preventable disease, HCC secondary to NAFLD should be considered as a preventable cancer.

Recent studies suggested that non-cirrhotic NAFLD patients with HCC have larger tumors at the time of diagnosis and the patients are older than cirrhotic patients, resulting in poorer prognosis [6], which emphasizes the importance of early diagnosis and treatment. The HCC in a non-cirrhotic patient population may have extrahepatic metastasis at the time of presentation, which prompts the urgent need of surveillance of HCC, particularly in patients with NAFLD. Therefore, five-year survival could be as high as 50–70% if HCC is diagnosed at an early stage and the treatment is aggressive with curative intent [7].

## Conclusions

Early recognition with effective surveillance and curative treatment could further increase the survival in HCC patients. However, there are no current guidelines for the surveillance of HCC in non-cirrhotic NAFLD patients. This warrants larger prospective studies to explore this important entity of NAFLD-induced HCC. The benefits of surveillance programs need to be determined, and such programs should be implemented if they are cost effective. In addition, clinicians should have a high index of suspicion for HCC while dealing with NAFLD patients with concerning clinical presentations regardless of whether they have full blown liver cirrhosis, minimal fibrosis, or no cirrhosis at all.
